# Wake up, College of Nursing! Further Signs of Awakening

**Published:** 1919-01-11

**Authors:** 


*To the Editor of* The Hospital.

### Wake up, College of Nursing! Further Signs of Awakening.

*To the Editor of* The Hospital.

Sir,—I have been interested to watch the gradual response to "Ierne's" clarion call for nurses to articulate, and feel that the next article should be entitled "The Awakened College," as letters from members in your correspondence columns indicate that it is awakened. I think, however, that we who belong to the College do not realise sufficiently how responsible we are for its progress and activities. In the first instance, we elect our representatives on the Council, and, secondly, it is by our efforts that the membership will be doubled and the College become, as a result, such a power that our demands, economic, educational, and all others, will receive a hearing. Herein, I believe, is the secret of the further achievements of the College, and it is a point which Miss Yapp emphasises in her letter this week, that a College made powerful by a large membership must automatically improve every aspect of the profession; and as I ponder this over, as I often do, I feel the Council is wise in hesitating to dictate reforms which would be justifiable from an authority which represents the greater part of the nursing profession. The fact that they have not as yet publicly dictated any definite policy with reference to the economic and educational programme may not mean, and I hope it does not, that they have no constructive schemes ready to launch as soon as they are convinced of the support of the rank and file, which can only be demonstrated by the strength of the College register. We members only, who are, after all, the College, are to blame if its development is slow.

We must hope, therefore, that the members who have learnt to articulate at the invitation of "Ierne" are not stopping at that, but are determined to make possible all they desire for our College by getting others to join us. And what of our responsibility in the selection of representatives? I suppose before very long we shall be receiving our nomination papers. Have we found the representative who has a definite policy and the time to work for it on the Council? I think I have found one, and I am going to ask my Centre's support of her candidature. Let us begin at once to take an active interest in the privilege granted to us by the College, in the use of the vote at the coming election.—I am, Sir, yours faithfully,

An Awakened Member.

